# Role of fluctuations in epidemic resurgence after a lockdown

**DOI:** 10.1038/s41598-021-85808-z

**Published:** 2021-03-19

**Authors:** I. Neri, L. Gammaitoni

**Affiliations:** NiPS Lab, Dipartimento di Fisica e Geologia, Università degli studi di Perugia, 06010 Perugia, Italy

**Keywords:** Complex networks, Statistical physics

## Abstract

Most popular statistical models in epidemic evolution focus on the dynamics of average relevant quantities and overlooks the role of small fluctuations on the model parameters. Models for Covid-19 are no exception. In this paper we show that the role of time-correlated fluctuations, far from being negligible, can in fact determine the spreading of an epidemic and, most importantly, the resurgence of the exponential diffusion in the presence of time-limited episodes in promiscuity behaviours. The results found in this work are not only relevant and specific for the Covid-19 epidemic but are more general and can be applied to other epidemics.

## Introduction

In present days of the Covid-19 epidemic dynamics, when the maximum of the infection spreading has passed in most western countries, there is a growing concern that time-limited episodes of large increases in promiscuity, might bring important resurgence in the spreading of the infection. We show that, in order to model the effect of such episodes it is of fundamental importance to take in duly account the unavoidable presence of fluctuations in the promiscuity behaviour. Neglecting the presence of time-correlated random fluctuations can lead to under-estimating the future evolution of the epidemics.

Although the need for a stochastic dynamics approach to epidemic modelling is well recognised^1–5^, the general tendency is to rely on a statistical approach where the role of fluctuations is accounted for through a probabilistic approach^6,7^ or by considering only white gaussian fluctuations^8,9^. In the following we will address the stochastic dynamics of the epidemics through a Langevin-based approach (i.e. through stochastic differential equations in the presence of time-correlated fluctuations) and will show how does this compare to the (average-parameter) deterministic description.

To fix our ideas, we will focus on the simple susceptible-infected-removed (SIR) model^10^ applied to a fixed population of *N* subjects. However, conclusions drawn here have a much general validity, specifically for the wide class of compartmental epidemic models.

## Results

As a realistic model we considered the population of the Umbria region, in Italy. There, the epidemic spread has reached its maximum approximately on April 5, 2020 (day 36)^11^ and has subsequently decreased with a total of removed $R=1400$ as of May 30 2020 (day 91). On March 13 (day 13) a lockdown was decided all-over the country and this affected the spread of the infection in Umbria, as well. The lockdown was subsequently gradually removed, starting on May 18 (day 65). According to the standard SIR model, we consider $N=820{,}000$ the total, fixed, population that, at any time *t*, is composed by $S(t)+I(t)+R(t)=N$. Here, *S*(*t*) indicates the number of healthy people at time *t*, that is susceptible to get infected. *I*(*t*) indicates the number of actually infected people and *R*(*t*) the number of the removed from *I*(*t*), i.e. the deceased plus the survived that cannot be infected again. The deterministic dynamics of the populations is expressed by a set of ordinary differential equations:

1 $$\begin{aligned} \left. \begin{aligned} \dot{S}(t)&= -\beta ~ {{S(t)I(t)}\over {N}} \\ \dot{I}(t)&= \beta ~ {{S(t)I(t)}\over {N}} - \gamma I(t) \\ \dot{R}(t)&= \gamma I(t) \end{aligned} \right. \end{aligned}$$

The relevant parameters of this dynamics are $\beta$ and $\gamma$, that represent the rate of passage from *S*(*t*) to *I*(*t*) and from *I*(*t*) to *R*(*t*), respectively. We can express $\beta$ as the product of two factors $\beta = C T$ where *C* represents the promiscuity, i.e. the tendency to socialise, to establish close contacts. The larger C the greater the number of contacts per day per person. On the other hand, *T* represents the capacity of the virus to be transmitted from person to person during a single contact. The larger is *T*, the greater is the probability to get infected in a single contact. Finally, $\gamma$ represents the removal rate, i.e. the probability to get out of the infected condition, due to healing or death. Public policies aimed at reducing the spread of the epidemics usually try to reduce the value of $\beta$ by affecting *C*, with quarantines and lockdown and *T* with protection masks and social distancing (through this paper we assume $\gamma = 0.037$, $T=0.43$).

**Figure 1:**
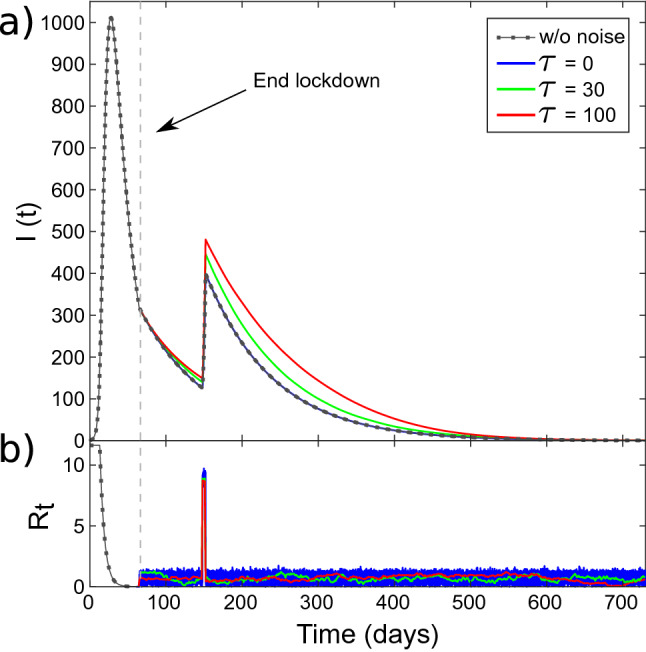
(**a**) Population *I*(*t*) from the numerical solution of the SIR model in (1). (**b**) Reproduction Number $$R_t $$ versus time. The lockdown phase is characterised by an exponential decrease in this parameter. The narrow, rectangle impulse at day 150 represents time-limited increase in the $$\beta $$ coefficient, due to a large gathering event. Dot-dashed lines represent *I*(*t*) and $$R_t$$ without any fluctuation, respectively in (**a**, **b**). Continuous lines represent the same quantities in the presence of fluctuations. $$\beta $$ si affected by noise with correlation time $$\tau = 0$$ days (blue), $$\tau = 30$$ days (green), $$\tau = 100$$ days (red). Here $$\sigma = 0.009$$ and $$\langle \xi \rangle = 0$$. The end of the lockdown phase is marked by the vertical dashed line.

In order to keep the epidemics under control, authorities use to monitor a parameter, the Reproduction Number^12^ $R_t = (\beta /\gamma ) (S(t)/N)$, associated with the rate of change *dI*(*t*)/*dt*. The epidemic growth is conditioned by a positive rate of change and this, according to the second equation in (1), implies $R_t > 1$. In the initial phase of the epidemics, where $S(t)/N \approx 1$, $R_0 = \beta / \gamma > 1$ represents the condition that starts the exponential growth phase. In general, if the numerical impact of the epidemics is small compared to the total population (like in the Umbria case), we can consider simply $R_t = \beta /\gamma$. In Fig. 1a we present the results of the model for the infected population *I*(*t*), based on the data from the Umbria region. We modelled the lockdown occurred between day 13 ($t_{start}$) and 65 ($t_{end}$), as an exponential damping of the promiscuity index *C*(*t*) for the duration of the event.

2 $$\begin{aligned} C(t) = {\left\{ \begin{array}{ll} C_0 &\quad 0< t < t_{start}\\ C_0 \exp (-t/\tau _0) &\quad t_{start} \le t \le t_{end}\\ C_{end} &\quad t > t_{end} \end{array}\right. } \end{aligned}$$

with $\tau _0 = 6$ days. $C_{0}$ and $C_{end}$ are chosen such that before the start and after the end of the lockdown we have $R_t = 11.6$ and $R_t = 0.7$, respectively. These values have been chosen to mimic the epidemic evolution in the Umbria region^11^.

In this phase of the epidemics, when, thanks to the social restrictions^13^, the *I*(*t*) curve has reached small values, there is a growing concern that changes in the population attitude might bring a resurgence of the growth, producing a second peak.

One potential risk is represented by the occasional gathering of people. These are intense, time-limited events, like public festivals (that span for a week) or civic or religious celebrations (one or two days) or public protests (few hours) that in a relatively small community, like in the Umbria region, they can easily interest up to $20\%$ of the population. If $\tau _e$ is the duration of the event, we can model it with an impulse-like additional contribution to the *C*(*t*) parameter (due to a sudden and time-limited increase in the promiscuity):

3 $$\begin{aligned} C_e(t) = A~ {\textit{rect}} \bigg ({{t-t_e}\over {\tau _e}} \bigg ) \end{aligned}$$

where the *rect* function indicates a rectangular impulse of amplitude *A* and width $\tau _e$ that starts at time $t_e=150$ days, with $t_e > t_{end}$. Thus, our promiscuity function, at the end of the lockdown period, now reads $C(t) = C_{end} + C_e(t)$ for $t > t_{end}$.

**Figure 2:**
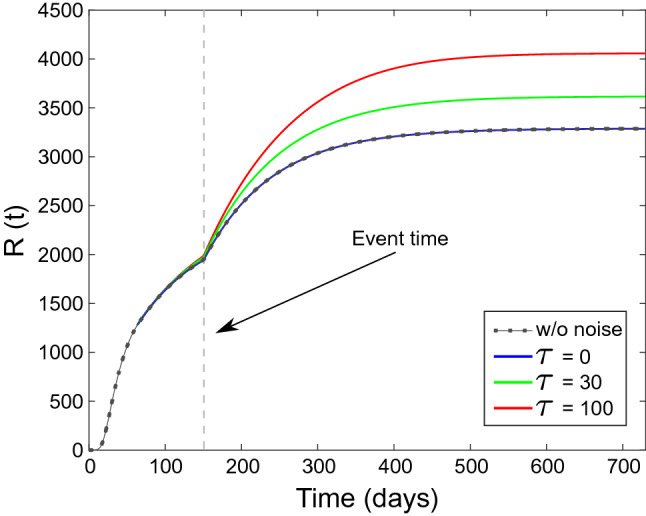
Population *R*(*t*) from numerical solution of (1). Dot-dashed line represents the population *R*(*t*) without any fluctuation in $\beta$. Continuous lines represent *R*(*t*) in the presence fluctuations. Noise correlation time $\tau = 0$ days (blue), $\tau = 30$ days (green), $\tau = 100$ days (red).

For there results presented in the curves of Figs. 1 and 2 we used $$A=0.7$$ and $$\tau _e = 4$$ days. We can observe that, although the event produces a significant increase in the Reproduction Number $$R_t $$ (Fig. 1b), its impact in the epidemic growth is quite limited (Fig. 1a, dotted curve). We notice that by solely monitoring the $$R_t$$ in this condition, might bring a sense of false security, due to the fact that low-amplitude and time-limited events seem to show limited consequences. This is also visible in Fig. 2, where we show the change in *R*(*t*) due to the presence of the event (Fig. 2, dotted curve).

However, as we are going to show, the impact of even such a limited event might be significantly larger if we take into account the role of fluctuations affecting the promiscuity attitudes. It is a fact that most of the popular approaches to epidemic modelling^6^, avoid taking into account small fluctuations and focus only on slow, deterministic changes in the *C*(*t*) (and thus $\beta$) parameter.

In order to show the potential impact of such fluctuations, we will assume that *C*(*t*) can be affected by noise, due to random changes on people behaviour from day-to-day practice. We do so by adding a stochastic signal $\sigma \xi (t)$, so that $C(t) = C_{end} + C_e(t) + \sigma \xi (t)$, with $\sigma = 0.009$ and $\xi (t)$ a gaussian distributed, zero average, unitary standard deviation, exponentially correlated noise, with correlation time $\tau$. As a consequence $\beta = T C(t)$ becomes a random variable and (1) a set of stochastic differential equations. By the moment that $\beta$ is a positive-defined quantity, we require that $C(t) \ge 0$.

In Figs. 1 and 2, we present the impact of an exponentially correlated noise on the populations *I*(*t*) and *R*(*t*), respectively, where we show the numerical solutions of (1) for three different values of the noise correlation time $\tau$. Although all the three cases have the same average ($\langle \xi \rangle =0$) and standard deviation $\sigma =0.009$, the change in the number of removed is remarkable.

Specifically, we observe that, for the delta-correlated noise ($\tau = 0$), the role of fluctuations is actually negligible and there is no increase in *I*(*t*) and *R*(*t*). However, increasing the correlation time $\tau$, we observe a significant increase in both the curves. This is apparent in the shape of the curve before the impulsive event and in the impact of the event itself.

**Figure 3:**
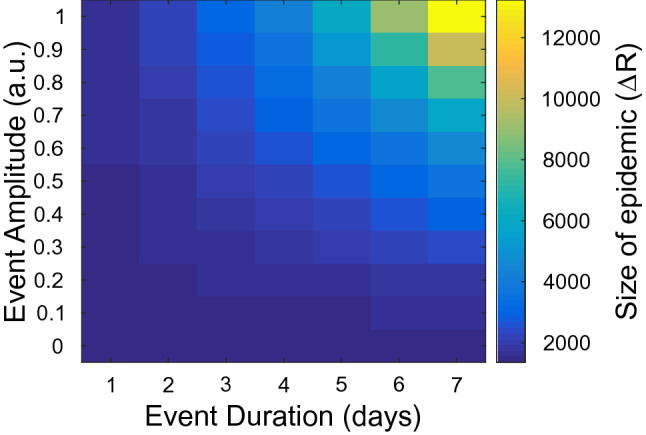
Impact of the impulse-like, time-limited event, in the presence of fluctuations, estimated by monitoring the change in the expected *size of the epidemic*, $\Delta R$, versus event duration ($\tau _e$) and intensity (*A*). $\tau = 100$ days. $\sigma$ and $\langle \xi \rangle$ as in Figs. 1 and 2.

To express quantitatively such an impact, we estimated the change in the so-called *size of the epidemic* $N - S(t_\infty ) = R(t_\infty )$. The change in this quantity, before and after the impulsive event, is expressed by $\Delta R = R(t_\infty ) - R(t_{end})$, where $t_\infty \gg t_e$ is a proper time, chosen when *R*(*t*) has reached the growth plateau. In Fig. 3, we show the value of $\Delta R$, for $\tau = 100$ days and a wide interval of $\tau _e$ and *A* values. As expected, $\Delta R$ grows when both $\tau _e$ and *A* grow.

**Figure 4:**
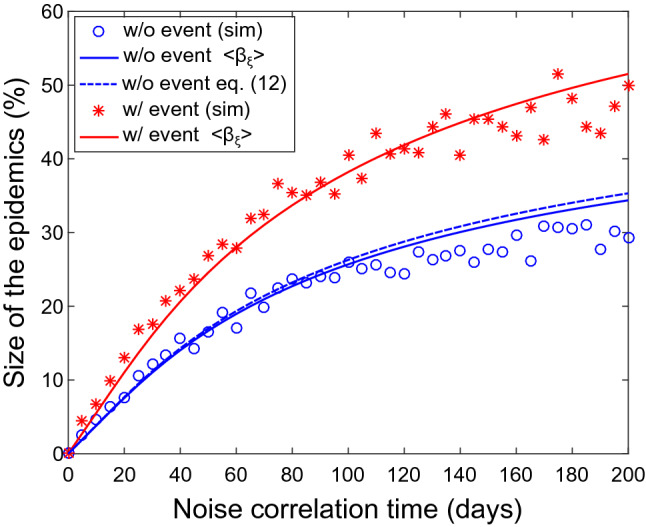
Size of the epidemics $\Delta R$ as a function of the noise correlation time $\tau$. Dots refer to numerical solution of (1) in the presence of fluctuations: (red) when an event with amplitude $A=0.7$ and duration $\tau _e = 4$ days is present and (blue) without any impulsive event. Continuous lines represent numerical solution of (1) with $\beta = \beta _d + \langle \beta _\xi \rangle$, according to (7). Blue dashed line represents the theoretical prediction in Eq. (12).

In order to account for these behaviours and provide a detailed modelling of the effect of noise, we studied the behaviour of the size of the epidemic change $\Delta R$ as a function of the noise correlation time $\tau$. In Fig. 4 we present the results of the digital simulation (dots) together with the prediction of our model (lines) expressed as relative changes. We notice that $\Delta R$ grows monotonically with noise correlation time. This is true both for the case without the time-limited event (lower curve) and with it (upper curve). The change in $\Delta R$, due to a highly correlated noise can be very significant, up to $50\%$ of the involved population. The role of white noise, instead, is negligible and the epidemic response is completely accounted for by the purely deterministic evolution.

All these feature can be accounted for by considering that the Reproduction Number $R_t$, in the presence of noise, can be expressed as a composition of the original (i.e. in the absence of noise) value plus the contribution of the fluctuating part:

4 $$\begin{aligned} R_t(t) = {\beta _d \over \gamma } + {\beta _\xi \over \gamma } \end{aligned}$$

with

5 $$\begin{aligned} \left. \begin{aligned} \beta _d&= T (C_{end} + C_e(t)) \\ \beta _\xi&= T \sigma \xi (t) \end{aligned} \right. \end{aligned}$$

By the moment that the noise $\xi (t)$ represents a zero-average contribution, the usual approach^6^ in these cases is to rule out the role of fluctuations, considering $\langle \beta _\xi \rangle =0$ and thus $\langle R_t(t)\rangle =\beta _d / \gamma$, and go back to the original set of equations (1) with $\langle S(t) \rangle = S(t)$, $\langle I(t) \rangle = I(t)$ and $\langle R(t) \rangle = R(t)$. As we have seen in Figs. 1 and 2, this is justified if the noise is white.

However, in the case of coloured noise, i.e. finite-time correlated fluctuations, the time-averaging operation has to be taken with some attention. In particular, in the case of averaging in a short (compared to the noise correlation time) time window, this might result in a non zero $\langle \beta _\xi \rangle$. In fact, this is the case if we consider that the relevant time window of the epidemics dynamics is represented here by the quantity $\Delta \approx 1/|(\beta - \gamma )|$. This is clear if we consider the time evolution of the infected *I*(*t*) in (1). In this case the $\tau$-correlated noise contribution $\langle \beta _\xi \rangle$, has to be computed through a moving average, with a time window of width $\Delta$. By the moment that time averaging can be interpreted as low-pass filtering, we represent the time averaging procedure in terms of a convolution operation between the auto-correlation function of the noise and the rectangular window ${\textit{rect}}(t/\Delta )$ with width $\Delta$:

6 $$\begin{aligned} \Gamma (t) = \int _{-\infty }^{\infty } {\textit{rect}} \bigg ( {{t}\over {\Delta }} \bigg ) e^{-{{t-\bar{t}}\over {\tau }}} d\bar{t} \end{aligned}$$

The time averaged contribution of the fluctuating $\beta _\xi$ can thus be expressed as $\langle \beta _\xi \rangle = \sigma \Gamma (\Delta /2)/\Delta$. Carrying out the finite integral $\Gamma (\Delta )$ we finally obtain:

7 $$\begin{aligned} \langle \beta _\xi \rangle = T \sigma {{\tau }\over {\Delta }} \left( 1-e^{-{\Delta \over {\tau }}}\right) \end{aligned}$$

It is important to note that this expression provides $\langle \beta _\xi \rangle$ only in implicit way, by the moment that

8 $$\begin{aligned} \Delta = {{1}\over {|(\beta - \gamma )|}} = {{1}\over {|(\beta _d + \langle \beta _\xi \rangle - \gamma )|}} \end{aligned}$$

that prevents from obtaining an analytic expression for $\langle \beta _\xi \rangle$. However Eq. (7) can be solved numerically. The solid lines in Fig. 4 represent the result of the numerical solution of the SIR model where $\beta = \beta _d + \langle \beta _\xi \rangle$ and $\langle \beta _\xi \rangle$ is provided by the numerical solution of Eq. (7).

In the approximation $S(t)/N \approx 1$, we can derive an analytic solution for $\Delta R$ that uses the second and third equations in (1). From the second equation we obtain:

9 $$\begin{aligned} I(t)= {k \over {\beta -\gamma }} ~ e^{(\beta -\gamma )t} \end{aligned}$$

where $I(0)=I_0 = k/(\beta - \gamma )$. Substituting Eq. (9) into the third equation and solving for *R*(*t*) we obtain:

10 $$\begin{aligned} R(t)= {{I_0 \gamma } \over {\beta -\gamma }} ~ \left( e^{(\beta -\gamma )t} - 1 \right) + R_0 \end{aligned}$$

We are interested in evaluating the impact in correspondence of the isolated event, thus we set $R_0 = R(t_{end})$. we have:

11 $$\begin{aligned} \Delta R(t)= {{I_0 \gamma } \over {\gamma -\beta }} ~ \left( 1- e^{-(\gamma -\beta )t} \right) \end{aligned}$$

and thus:

12 $$\begin{aligned} \Delta R(t_{\infty }-t_{end})= {{I_0 \gamma } \over {\gamma -\beta }} \end{aligned}$$

This quantity is also presented in Fig. 4 (dashed curve). As we can see the theoretical curve follows closely the numerical solution, in good agreement with the results of the stochastic simulation.

## Discussion

In conclusion, we discussed the role of fluctuations in epidemics dynamics, with special attention to the impact that impulsive, time-limited events, may have on the resurgence of the epidemic growth, after the lockdown phase. Specifically we have shown that the role of even zero-averaged, small-amplitude random fluctuations, with correlation time of the order of, or longer than, the characteristic time scale of the epidemics dynamics, results in a significant amplification of the size of the epidemics. We presented a model to quantitatively estimate the impact of such fluctuations on the effective Reproduction Number $R_t$ and on the size of the epidemics $\Delta R$. Neglecting the role of fluctuations might result in an underestimation of $R_t$ with potential consequences on the size of the epidemics itself.
